# Mitochondrial DNA Copy Number Is Associated with Breast Cancer Risk

**DOI:** 10.1371/journal.pone.0065968

**Published:** 2013-06-12

**Authors:** Bharat Thyagarajan, Renwei Wang, Heather Nelson, Helene Barcelo, Woon-Puay Koh, Jian-Min Yuan

**Affiliations:** 1 Department of Laboratory Medicine and Pathology, University of Minnesota, Minneapolis, Minnesota, United States of America; 2 University of Pittsburgh Cancer Institute, Pittsburgh, Pennsylvania, United States of America; 3 Masonic Cancer Center and Department of Epidemiology and Community Health, University of Minnesota, Minneapolis, Minnesota, United States of America; 4 Duke-NUS Graduate Medical School Singapore, Singapore, Singapore; 5 Saw Swee Hock School of Public Health, National University of Singapore, Singapore, Singapore; 6 Department of Epidemiology, Graduate School of Public Health, University of Pittsburgh, Pittsburgh, Pennsylvania, United States of America; University of Texas Health Science Center at San Antonio, United States of America

## Abstract

Mitochondrial DNA (mtDNA) copy number in peripheral blood is associated with increased risk of several cancers. However, data from prospective studies on mtDNA copy number and breast cancer risk are lacking. We evaluated the association between mtDNA copy number in peripheral blood and breast cancer risk in a nested case-control study of 183 breast cancer cases with pre-diagnostic blood samples and 529 individually matched controls among participants of the Singapore Chinese Health Study. The mtDNA copy number was measured using real time PCR. Conditional logistic regression analyses showed that there was an overall positive association between mtDNA copy number and breast cancer risk (P_trend_ = 0.01). The elevated risk for higher mtDNA copy numbers was primarily seen for women with <3 years between blood draw and cancer diagnosis; ORs (95% CIs) for 2^nd^, 3^rd^, 4^th^, and 5^th^ quintile of mtDNA copy number were 1.52 (0.61, 3.82), 2.52 (1.03, 6.12), 3.12 (1.31, 7.43), and 3.06 (1.25, 7.47), respectively, compared with the 1^st^ quintile (P_trend_ = 0.004). There was no association between mtDNA copy number and breast cancer risk among women who donated a blood sample ≥3 years before breast cancer diagnosis (P_trend_ = 0.41). This study supports a prospective association between increased mtDNA copy number and breast cancer risk that is dependent on the time interval between blood collection and breast cancer diagnosis. Future studies are warranted to confirm these findings and to elucidate the biological role of mtDNA copy number in breast cancer risk.

## Introduction

Breast cancer is the most common cancer among women in the United States and accounts for 29% of all cancers in women with approximately 226,870 new cases expected to occur in 2012 [1]. The incidence of breast cancer is also rapidly increasing in several Asian populations and breast cancer is one of the most frequently diagnosed cancers in women worldwide [2]. Although early detection using mammography and improved treatments for invasive breast cancers have significantly reduced morbidity and mortality due to breast cancer, the malignancy remains the second leading cause of cancer death among women in the United States [1] and remains an important cause of cancer related deaths in women around the world [2].

Mitochondria, whose principal function is to generate energy through aerobic respiration, are the major source and target of intracellular reactive oxygen species (ROS) that plays an important role in breast carcinogenesis [3], [4]. Each cell has multiple copies of mitochondria and each mitochondrion has 2–10 copies of the mitochondrial genome [5], [6]. The amount of mitochondrial DNA (mtDNA) remains relatively stable within the cells under physiological conditions [5], [7]. As reviewed by Radppour et al, several studies have found somatic mitochondrial mutations and deletions in breast tumor tissue, suggesting that changes in mtDNA may play a significant role in breast carcinogenesis [8]. This hypothesis has been supported by epidemiological studies that demonstrated a statistically significant association between increasing mtDNA copy number in peripheral blood and increased risk of breast cancer [9] as well as other malignancies including Non-Hodgkin lymphoma [10], lung cancer [11], pancreatic cancer [12], and colorectal cancer [13], [14]. However, other studies showed different results. A retrospective case-control study showed decreased mtDNA copy number associated with increased breast cancer risk [15]. One recent prospective study found no association between mtDNA copy number and gastric cancer [16] while two retrospective case-control studies showed an increased risk of renal cancer associated with decreased mtDNA copy number [17], [18]. Given that the development of and treatment for cancer can cause oxidative stress, which could impact mtDNA copy number, a prospectively designed study with blood samples collected many years prior to cancer diagnosis could overcome this potential problem. Therefore we conducted a nested case-control study within the Singapore Chinese Health Study (SCHS) using blood samples that were collected from the cohort participants before breast cancer diagnosis. The present study allowed us to evaluate the association between mtDNA copy number and the risk of breast cancer in pre-diagnostic peripheral blood samples.

## Materials and Methods

### Study Population

The study participants were chosen from the Singapore Chinese Health Study (SCHS), a population-based prospective cohort of 63,257 Chinese women and men from two major dialect groups, the Hokkiens and Cantonese, aged 45–74 years who were enrolled in the study from April 1993 through December 1998 [19]. The participants lived in government housing estates that were home to 86% of the Singapore population. At recruitment, a trained interviewer used a structured questionnaire to conduct a face-to-face interview in the participant’s home and collected information on demographics, tobacco use, physical activity, menstrual/reproductive history in women, medical history, and family history of cancer. A 165-item food frequency questionnaire was validated against a series of 24-hour dietary recall interviews was used to collect information on diet and alcohol use [19].

Blood and single-void urine specimens were requested from subjects drawn from a 3% random sample of enrollees in April 1994. All surviving members of the cohort were contacted to provide a biospecimen between January 2000 and April 2005 and biospecimens were obtained from more than 32,500 participants. This study was approved by the Institutional Review Boards of the National University of Singapore, University of Pittsburgh, and the University of Minnesota and written informed consent was obtained from all participants.

*Cases:* The population-based cancer registry in Singapore was used to identify the incident breast cancer cases [20]. Only cohort participants who donated a blood sample between January 2000 and April 2005 and developed breast cancer as of December 31, 2007 were included in the present study. In total, 183 breast cancer patients, whose diagnosis were confirmed by manual reviews of clinical/pathology reports and who provided a blood sample before their development of breast cancer were included in the present analysis.

*Controls:* For each case, 3 control women were randomly selected among the cohort participants of the Singapore Chinese Health Study who donated a blood sample between January 2000 and April 2005 and were alive and remained free of cancer on the date of breast cancer diagnosis of the index case, The control women were individually matched with the breast cancer cases with respect to age at baseline interviews (±3 years), date of baseline interview (±2 years), dialect group (Cantonese vs. Hokkien), menopausal status at sample collection and date of biospecimen collection (±6 months). In total, 529 women who met these criteria were chosen as a comparison control group.

### Laboratory Methods

All DNA samples were extracted from 0.5 ml of buffy coat fraction of the whole blood using the QiaAMP 96 DNA blood kits (Qiagen Inc., Gaithersburg, MD, USA). DNA samples of 183 breast cancers and 529 individually matched controls were retrieved from the biorepository of the Singapore Chinese Health Study. All DNA samples of a given matched set (containing the samples from the case and the three matched controls) were arranged in random order, identified only by unique codes, and tested in the same batch of laboratory assay for mitochondrial DNA (mtDNA) copy number. The details of the mtDNA copy number assay have been described previously [14]. MtDNA copy number was measured using a real time quantitative polymerase chain reaction (PCR) using an Applied Biosystems 7900 Sequence Detection System (Applied Biosystems, Foster City, CA). One primer pair specific for the mitochondrial DNA (*ND1*) and another primer pair specific for the nuclear DNA (*18s*) were designed for relative quantification for mtDNA copy number. The primer sequences for the mitochondrial *ND1* gene were: forward primer (ND1-F), 5′- CCCTAAAACCCGCCACATCT-3′; reverse primer (ND1-R), 5′-GAGCGATGGTGAGAGCTAAGGT-3′. The primer pair used for the amplification of the nuclear gene *18s* was as follows: forward primer (18s-F), 5′-TAGAGGGACAAGTGGCGTTC-3′; reverse primer (18s-R), 5′-CGCTGAGCCAGTCAGTGT-3′. Standard curves made by serial dilution of a reference DNA sample was used to determine the ratio of mtDNA copy number to the amount of nuclear DNA that is proportional to the mtDNA copy number in each cell. All samples were assayed in duplicate. A calibrator DNA (i.e. genomic DNA from a healthy control volunteer) was used to standardize analytical variation between different mtDNA copy number assay runs. The 14 µL PCR mixture contained 1× SYBR Green mastermix (Applied Biosystems; Foster City, CA, USA), 215 nM ND1-R (or 18s-R) primer, 215 nM ND1-F (or 18s-F) primer, and 0.4 ng of genomic DNA for *ND1* and *18s*. The thermal cycling conditions were 95°C for 10 minutes, followed by 40 cycles of 95°C for 15 seconds, and 60°C for 1 minute for *ND1* and 62°C for 1 minute for *18s*. The efficiency of all quantitative PCR runs ranged from 99% to 110%.The R^2^ for all standard curves was ≥0.99. Standard deviations for the cycle of threshold (Ct) duplicates were ≤0.25. Based on analyses of 27 blinded duplicate samples analyzed on two different days the coefficient of variation (CV) was 14%.

### Statistical Analyses

All statistical analyses were carried out using SAS software version 9.1.3 (SAS Institute, Cary, NC). The distribution of the relative mtDNA copy number (i.e. the ratio of mtDNA to nuclear DNA in the study samples normalized to a reference DNA sample) was markedly skewed toward high values and corrected to a large extent by transformation to logarithmic values. Therefore, all statistical tests were performed on logarithmically transformed values, and geometric means are presented. The χ^2^ test and the *t*-test were used to compare the distributions of demographics, lifestyle and reproductive characteristics between breast cancer cases and controls. Pearson correlation coefficient was used to evaluate the correlation between mtDNA copy number and time interval between blood collection and breast cancer diagnosis. Differences in the relative mtDNA copy number across different groups of selected reproductive characteristics among control participants were examined using analysis of covariance (ANCOVA). These analyses were adjusted for age and analytical run in which mtDNA copy number was measured (batch number) to control for variation in relative mtDNA copy number across different analytical runs. Conditional logistic regression models retaining original matched sets that controlled for matching factors and the laboratory batches for mtDNA assays were used to evaluate the association between relative mtDNA copy number and breast cancer risk after adjustment for body mass index (BMI), age at menarche and number of live birth. The study participants were grouped into quintiles according to the distribution of relative mtDNA copy number among control subjects. Additional subgroup analyses for the mtDNA-breast cancer risk were performed using conditional logistic regression models according to the time interval between blood sample collection and breast cancer diagnosis (≥3 years vs. <3 years) and breast cancer stage at the time of diagnosis. All *P*-values <0.05 were considered to be statistically significant.

## Results

Women with breast cancer were more likely to be nulliparous or have fewer live births as compared to control women (Table 1). Compared to controls, cases were also more educated, had earlier age at menarche (≤14 years), and more likely to have BMI ≥28 kg/m^2^ or use hormone replacement therapy, although these differences did not reach statistical significance (Table 1). The relative mtDNA copy number was not associated with any of the reproductive variables such as age at menarche, menopausal status, use of hormone replacement therapy and number of live births (p>0.05) among control women (Table 2).

**Table 1:** Demographic and reproductive characteristics of breast cancer patients and control women, the Singapore Chinese Health Study.

	Cases (%)	Controls (%)	*P*
	(n = 183)	(n = 529)	
**Age at sample collection** (mean ± SD)	61.1±8.3	61.1±8.1	0.96
**Dialect group**			
Cantonese	54.6	53.7	
Hokkien	45.4	46.3	0.82
**Highest level of education**			
No formal education	22.4	30.4	
Primary school	47.5	41.8	
Secondary school or higher	30.1	27.8	0.11
**Body mass index (kg/m^2^)**			
<20	10.9	12.5	
20 to <24	50.3	52.2	
24 to <28	26.8	27.6	
28+	12.0	7.7	0.36
**Age at menarche (years)**			
<13	14.2	17.4	
13–14	48.6	37.6	
15–16	29.5	34.6	
17+/never became regular	7.7	10.4	0.07
**Number of live births**			
0	12.6	7.2	
1–2	36.1	30.0	
3–4	30.0	41.4	
5+	21.3	21.4	0.01
**Menopause status at sample collection**			
Pre-menopause	11.5	11.0	
Post-menopause	88.5	89.0	0.85
**Use of hormone replacement therapy**			
No	92.4	93.8	
Yes	7.6	6.2	0.51
**Weekly vigorous or strenuous physical activity**			
No	94.0	94.1	
Yes	6.0	5.9	0.94
**Family history of breast cancer** *			
No	98.4	98.5	
Yes	1.6	1.5	0.90

* Family history of breast cancer among first degree relatives.

**Table 2:** Geometric means of relative mtDNA number by selected reproductive variables among controls only, the Singapore Chinese Health Study.

	No	Mean* (95% CI)	*P*
Age at menarche (years)			
<13	92	2.18 (1.94, 2.44)	
13–14	199	2.12 (1.98, 2.30)	
15–16	183	2.10 (1.94, 2.26)	
17+/never became regular	55	2.12 (1.84, 2.46)	0.68
Number of live births			
0	38	2.04 (1.72, 2.42)	
1–2	159	2.14 (1.96, 2.34)	
3–4	219	2.18 (2.02, 2.32)	
5+	113	2.04 (1.82, 2.26)	0.87
Menopause status at sample collection			
Pre-menopause	58	2.14 (1.84, 2.48)	
Post-menopause	471	2.12 (2.02, 2.24)	0.91
Use of hormone replacement therapy			
No	496	2.12 (2.02, 2.22)	
Yes	33	2.22 (1.86, 2.66)	0.59

* Adjusted for age at sample collection and batch number of laboratory assays.

After adjustment for age at menarche, BMI and number of live births, relative mtDNA copy number was positively associated with breast cancer risk overall (p for trend = 0.01) (Table 3). Additional analyses evaluating the association between the relative mtDNA copy number and risk of incident breast cancer stratified by time interval between blood sample collection and breast cancer diagnosis showed that relative mtDNA copy number was associated with breast cancer risk only among those women from whom a blood sample was collected within 3 years of breast cancer diagnosis. Relative mtDNA copy number was negatively correlated with time to breast cancer diagnosis (r = −0.15; p = 0.048). Among women who donated a blood sample <3 years prior to breast cancer diagnosis, ORs (95% CIs) of breast cancer for women in the 2^nd^, 3^rd^, 4^th^, and 5^th^ quintiles of mtDNA copy were 1.52 (0.61, 3.82), 2.52 (1.03, 6.12), 3.12 (1.31, 7.43), and 3.06 (1.25, 7.47), respectively, compared with the 1^st^ quintile (p for trend = 0.004) (Table 3). In contrast, there was no association between mtDNA copy number and breast cancer risk among women who donated a blood sample ≥3 years prior to breast cancer diagnosis (p for trend = 0.41) (Table 3).

**Table 3:** The relative mtDNA copy number in relation to risk of breast cancer, the Singapore Chinese Health Study.

Time interval between blood draw and cancer diagnosis		mtDNA copy number in quintiles					
		1^st^ quintile	2^nd^ quintile	3^rd^ quintile	4^th^ quintile	5^th^ quintile	
		(<1.50)	(1.50–1.94)	(1.95–2.51)	(2.52–3.38)	(>3.38)	P for trend
All subjects	Cases/Controls	29/117	27/109	38/95	46/102	43/106	
	OR† (95%CI)	1.00	1.03 (0.56, 1.90)	1.70 (0.96, 3.01)	1.96 (1.12, 3.44)	1.75 (0.97, 3.15)	0.010
<3 years‡	Cases/Controls	11/63	13/50	17/41	24/51	23/47	
	OR† (95%CI)	1.00	1.52 (0.61, 3.82)	2.52 (1.03, 6.12)	3.12 (1.31, 7.43)	3.06 (1.25, 7.47)	0.004
3+ years‡	Cases/Controls	18/54	14/59	21/54	22/51	20/59	
	OR† (95%CI)	1.00	0.74 (0.32,1.73)	1.24 (0.58, 2.65)	1.41 (0.66, 2.99)	1.09 (0.48, 2.46)	0.405

† Odds ratios were calculated using conditional logistic regression models with adjustments for BMI, age at menarche, and number of live births.

‡ For all breast cancer cases, blood samples were collected 3.4 (SD = 2.3) years prior to cancer diagnosis.

## Discussion

This is the first study to demonstrate a prospective association between increased relative mtDNA copy number and increased breast cancer risk. The findings from the current study are in agreement with the findings from an earlier retrospective case-control study (100 breast cancer cases and 100 controls in a predominantly white population) [9]. However, these results are contradictory to another case-control study (60 breast cancer cases and 50 controls in a Chinese population) that showed decreased mtDNA copy number was associated with increased breast cancer risk [15]. Several other studies have shown mixed associations between mtDNA copy number and risk of several other cancers. One retrospective case-control study has shown an association between increased mtDNA copy number and increased risk of colorectal cancer [13] while other retrospective studies have shown an association between decreased mtDNA copy number in peripheral blood and increased risk of renal cancers [17], [18]. Three prospective studies from the Alpha-tocopherol, beta-carotene and cancer prevention (ATBC) study showed increased mtDNA copy number to be associated with increased risk of cancers in the lung [11], pancreas [12] and NHL [10]. Another prospective study from the Shanghai women’s cohort showed no association between mtDNA copy number and gastric cancer [16] while the Singapore Chinese Health Study showed a U shaped association between mtDNA copy number and colorectal cancer risk [14].

This study found that mtDNA copy number was negatively correlated with the time between blood draw and breast cancer diagnosis, and that the association between relative mtDNA copy number and breast cancer risk was restricted only to those women who donated a blood sample within 3 years of breast cancer diagnosis. A previous case-control study by Xia et al showed the stage I breast cancers had a lower mtDNA copy number in peripheral blood while stage II–IV breast cancers had increased mtDNA copy number in peripheral blood [15]. This finding along with the discrepant association between mtDNA copy number in peripheral blood and breast cancer risk observed in the two case-control studies suggest that breast cancer itself may modify that levels of mtDNA observed in peripheral blood and highlights the need for prospective study designs, such as the present study, to evaluate the association between mtDNA copy number in peripheral blood and breast cancer risk. Since mtDNA copy number is not associated with breast cancer risk among women who donated a blood sample 3 years prior to their breast cancer diagnosis and mtDNA copy number decreased with increasing time interval between dates of blood draw and breast cancer diagnosis, one possible explanation is that subclinical progression of breast cancer would have an impact on mtDNA copy number. The association between altered mtDNA copy in blood samples collected within 2 years of gastric cancer was also observed in the Shanghai women’s cohort though in that study lower mtDNA copy number among participants who donated a blood sample within 2 years of cancer diagnosis was associated with gastric cancer risk [16]. These findings further support the idea that both cancer stage and cancer site may influence mtDNA copy number in peripheral blood and, in prospective studies, these associations may be dependent on the timing of blood collection in regards to cancer diagnosis.

Several studies examined mtDNA copy number in tumor tissues as well as adjacent normal tissues of various organs. Increased mtDNA copy number was observed in tumor tissues of the colorectum [21], [22], lung [22], ovary [23], endometrium [24] and thyroid [25] whereas reduced mtDNA copy number were observed in tumor tissues of the breast [25], [26], stomach [22], [27], liver [22], [28] and kidney [29], as compared to their normal adjacent tissues. These data suggest that the role of tumor mtDNA in carcinogenesis might be different in different organs or tissue origin. Reduced mtDNA in tumors has been associated with somatic mitochondrial mutations [30] and mutations in nuclear genes such as p53 [31], which occur frequently in breast cancer. Thus the reduced mtDNA in breast tumors is likely a consequence of several tumor specific characteristics such as somatic changes in nuclear and mitochondrial genes and the increased endogenous oxidative damage observed in breast tumors though the exact mechanisms still remain incompletely understood [32]. In addition, reduced mtDNA copy number in breast tumors has also been associated with increased nuclear damage [33], poorer overall and disease free survival [30] and better response to anthracycline based therapy [34] suggesting a functional role for mitochondrial alterations in breast carcinogenesis. Additional studies are warranted to elucidate the organ-specific role of mtDNA in the development of cancer in humans.

The biological mechanism for increased relative mtDNA copy number in peripheral blood with risk of breast cancer is not completely understood. Since breast tumor specific mtDNA mutations have been rarely detected in peripheral blood [35], it is likely that the contribution of tumor specific mutations in determining mtDNA copy number in peripheral blood is minimal. Furthermore, since the correlation between mtDNA copy number in peripheral blood and breast tissue has not been evaluated in this study or previous studies, the biological relevance of increased mtDNA copy number in peripheral blood to breast carcinogenesis remains unclear. A positive association between the relative mtDNA copy number and markers of oxidative stress including thiobarbituric acid reactive substances and 8-hydroxyguanosine was observed in humans [36]. Lower levels of antioxidants in plasma were also associated with increased mtDNA copy number [36]. Fibroblasts that were exposed to mild oxidative stress show an increase in mitochondrial mass through a cell-cycle independent pathway [37]. High relative mtDNA copy number present in aging cells is thought to be the result of compensatory response to the cumulative exposures to oxidative stress and cumulative accumulation of mitochondrial DNA mutations over time [4]. Increased mtDNA copy number in peripheral blood may reflect increased levels of oxidative damage that has been associated with breast cancer risk [38], [39]. Thus mtDNA copy numbers in breast tumors and peripheral blood are influenced by biologically distinct mechanisms and may represent different biological processes that are relevant to breast carcinogenesis.

The strengths of the current study include comprehensive recording of breast cancer cases via the Singapore cancer registry since 1968 [40] and a relatively uniform access to specialized health care thereby providing complete ascertainment of breast cancer cases. The study participants originated from two contiguous regions in South China, leading to a high degree of genetic homogeneity. All reproductive and lifestyle factors were assessed prospectively and less likely to be influenced by recall bias. A relatively large sample size provided sufficient statistical power (80% power at α = 0.05 to detect an odds ratio of 2.0 between the highest quintile and the lowest quintile of relative mtDNA copy number) to examine the association between the relative mtDNA copy number and breast cancer risk. This is the first prospectively designed study to show a positive association between the relative mtDNA copy number and risk of breast cancer. Future studies are warranted to evaluate aging and oxidative stress related factors that influence mtDNA copy number and to provide novel insights into biological mechanisms of mtDNA copy number variation on the development of breast cancer.
